# Research Progress on the Relationship between Vitamins and Diabetes: Systematic Review

**DOI:** 10.3390/ijms242216371

**Published:** 2023-11-15

**Authors:** Jiameng Liu, Luqi Qin, Jiahuan Zheng, Litao Tong, Wei Lu, Cong Lu, Jing Sun, Bei Fan, Fengzhong Wang

**Affiliations:** Key Laboratory of Agro-Products Quality and Safety Control in Storage and Transport Process, Ministry of Agriculture and Rural Affairs, Institute of Food Science and Technology, Chinese Academy of Agricultural Sciences, Beijing 100193, China

**Keywords:** vitamins, diabetes, treatment, mechanism

## Abstract

Diabetes is a serious chronic metabolic disease that causes complications over time, bringing serious public health challenges that affect different countries across the world. The current clinical drugs for diabetes may lead to adverse effects such as hypoglycemia and liver and abdominal distension and pain, which prompt people to explore new treatments for diabetes without side effects. The research objective of this review article is to systematically review studies on vitamins and diabetes and to explain their possible mechanism of action, as well as to assess the role of vitamins as drugs for the prevention and treatment of diabetes. To achieve our objective, we searched scientific databases in PubMed Central, Medline databases and Web of Science for articles, using “vitamin” and “diabetes” as key words. The results of numerous scientific investigations revealed that vitamin levels were decreased in humans and animals with diabetes, and vitamins show promise for the prevention and/or control of diabetes through anti-inflammation, antioxidation and the regulation of lipid metabolism. However, a few studies showed that vitamins had no positive effect on the development of diabetes. Currently, studies on vitamins in the treatment of diabetes are still very limited, and there are no clinical data to clarify the dose–effect relationship between vitamins and diabetes; therefore, vitamins are not recommended as routine drugs for the treatment of diabetes. However, we still emphasize the great potential of vitamins in the prevention and treatment of diabetes, and higher quality studies are needed in the future to reveal the role of vitamins in the development of diabetes.

## 1. Introduction

Diabetes is a chronic metabolic disorder syndrome that is characterized by hyperglycemia and insulin resistance [1]. It is not only a severe driver of chronic metabolic diseases such as cardiovascular diseases (CVDs); it is also associated with many complications, such as diabetic kidney disease, diabetic retinopathy and diabetic neuropathy [2]. Although people have paid enough attention to the treatment of diabetes, its incidence has remained high in recent years. The latest edition of the International Diabetes Federation (IDF) Diabetes Atlas indicates that, if diabetes continues to be poorly treated, the amount of people with diabetes will grow rapidly to 693 million, accounting for 9.9% of the world’s population by 2040 [3].

Depending on the causes, symptoms and treatment, diabetes is generally divided into three categories: type 1 diabetes (TIDM), type 2 diabetes (T2DM) and gestational diabetes mellitus (GDM). T1DM is considered to be a class of autoimmune diseases for which the hallmark feature is to elevate blood glucose concentrations due to the loss of insulin-producing pancreatic β-cells. T2DM, one of the most common metabolic disorders, is caused by a combination of two primary factors: defective insulin secretion by pancreatic β-cells and the inability of insulin-sensitive tissues to respond appropriately to insulin. However, the cause of T2DM is complex and related to genetics and environment. GDM is an abnormal situation during pregnancy since the hormones made by the placenta prevent the body from using insulin effectively, it usual returns to normal after delivery [4]. At present, the management of diabetes involves the use of pharmacological agents such as oral hyperglycemia drugs and insulin injections. However, such treatments show limited efficacy, with longitudinal studies demonstrating that they would lose activity on glycemic control over time and may result in adverse effects, such as hypoglycemia and weight gain [5]. The limitations of current pharmacological treatments have prompted scientists to study the mechanism of diabetes and explore novel drugs. Evidence from epidemiological studies has shown that people with diabetes tend to have lower levels of micronutrients than healthy people; this finding has led to the renewed consideration of and focus on the relationship between micronutrients and diabetes. It has been reported that, when the level of 25(OH)VD in the blood is lower than normal, this results in the pancreatic β-cells being damaged [6]. Furthermore, the finding of vitamin D receptors on pancreatic β-cells has also promoted searches into the role of vitamins in the pathogenesis of the disease [7]. It is generally believed that the occurrence of T2DM is caused by the inflammatory stimulation. Therefore, it is reasonable that vitamin C, as a well-known antioxidant nutrient, can relieve the symptoms of diabetes by reducing the level of oxidative stress in the human body [8]. Vitamin A and its metabolites have been shown to play important roles in the regulation of glucose and lipid metabolism, implying VA’s potential role in the prevention and treatment of diabetes [9].

Although many studies have found an association between micronutrients and diabetes, it is not yet clear how vitamins affect diabetes, and whether micronutrient deficiency is a cause or a consequence of diabetes is still unknown. The aim of this review, therefore, is to collect the evidence surrounding the role of vitamin supplements in the management of diabetes and summarize the pathways of action that help guide current clinical practice.

## 2. Methods

### Data Sources and Data Extraction

**Figure 1:**
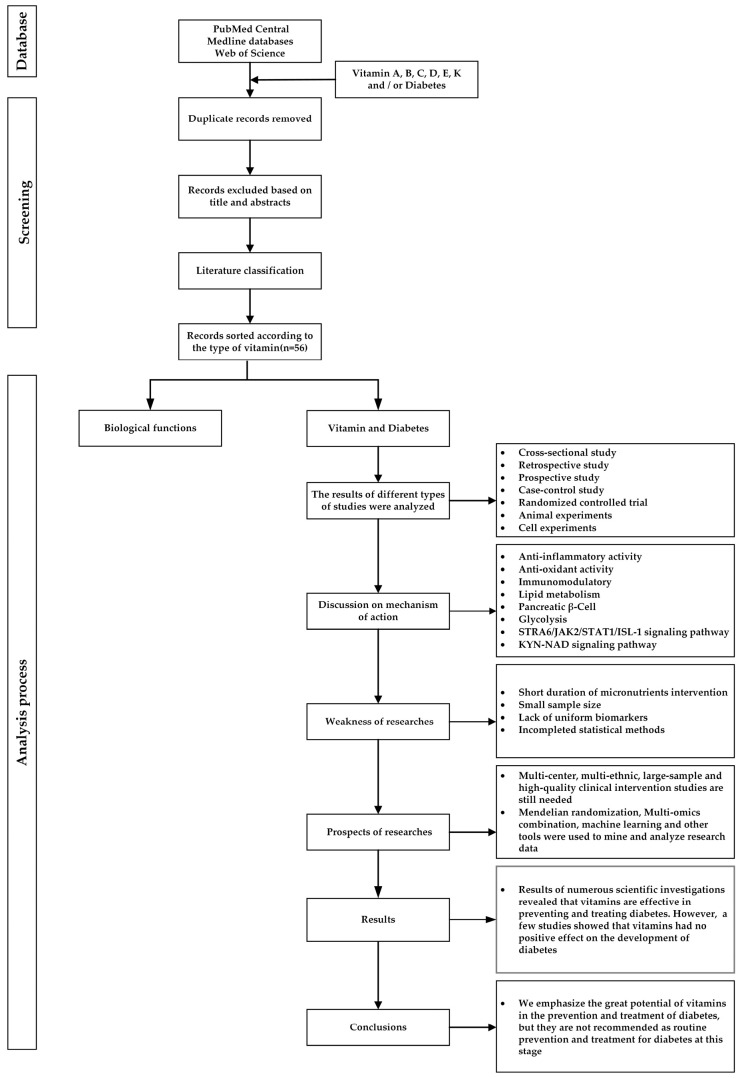
The literature screening and research process of vitamins and diabetes.

Systematic searches of the literature were conducted to locate peer-reviewed publications, using key terms including vitamins A–D and K, diabetes mellitus and the prevention and treatment of diabetes mellitus. The literature search was performed in the databases PubMed Central, Medline databases and Web of Science. Only peer-reviewed articles in which the titles and abstracts involved the previously listed key terms were selected. Publication selection included articles available from 1980 to 2023. (The research process is shown in Figure 1, and the literature is summarized in Table 1.)

## 3. Results and Discussion

### 3.1. Vitamin A

#### 3.1.1. Biological Functions

Vitamin A (VA) is the first micronutrient to be discovered. VA refers to all compounds with the biological activity of retinol. As an essential nutrient, VA is important for cell division and organ and skeletal growth and maturation [9]. VA deficiency has several adverse consequences at different stages of life. Generally, infants require increased VA to support rapid growth and help combat infections. During childhood, mild VA deficiency leads to increased risk and severity of infectious disease morbidity, possibly by altering the structure and function of the immune system. Severe VA deficiency can affect a person throughout his/her whole life by leading to night blindness, anemia and weakened resistance to infections [57]. There are two dietary sources of VA supplementation, one being retinol, which can be obtained directly from animal foods, including dairy products, liver and fish oil; the other is carotenoids, which are widely found in plant foods, which can be converted into retinol in vivo, including β-carotene, α-carotene, etc. [15]. The link between VA and metabolism was first discovered from observational studies of liver biopsies that T2DM patients showed two times-higher levels of hepatic VA content compared to healthy individuals [58]. Subsequently, the association between VA and diabetes was evaluated in different countries.

#### 3.1.2. Vitamin A and Diabetes

The Association of Nutrients and Epidemiology study in the United States found an inverse association between β-carotene intake and the risk of diabetes, and similar results were found in a large cohort study in the Netherlands involving 37,846 people [10,11]. However, the studies from Europe and United States found that there was no significant association [12,13]. The studies were conducted in American and European populations, whose diet is characterized by higher consumption of animal products and refined grains, in conjunction with lower consumption of vegetables and fruits. The same contradiction arises in Eastern populations with plant-based diets; for example, a prospective cohort study in Japan reported the relation between fat-soluble vitamins and the risk of T2DM in 19,168 healthy Japanese people aged 40-to-79 years old, and the results show that dietary VK and E were inversely associated with diabetes, but there was no significant association with dietary VA or VD [14]. In contrast to the Japanese study, an epidemiological study in China that lasted 26 years found that higher dietary intakes of total vitamins were associated with a lower risk of diabetes in both men and women, and dietary retinol intake was inversely related to a diabetes risk in men. The author argued that there may be a ceiling effect of total VA on the prevention of diabetes because the inverse association between VA intake and diabetes risk was only significant in the Chinese population with low VA intake, which was much lower than the VA intake in Japanese subjects [15].

Many studies have attempted to explain the possible mechanisms of how VA affects the development of diabetes. As mentioned above, VA shows an influence on cell development, including pancreatic cells, meaning that it is useful for the production of insulin. Animal studies showed that VA deficiency could lead to the loss of pancreatic β-cell mass and reduced insulin secretion, thereby causing hyperglycemia by inducing β-cell apoptosis [16,17]. On the other hand, retinol-binding protein 4 (RBP4), the primary circulating retinol transporter that delivers retinol from the liver to peripheral target tissues, had been found to have a positive U-shaped correlation between its level and the risk of T2DM, with the lowest risk in the range of 31 to 55 ug/mL RBP4 [18]. RBP4, which plays a previously unrecognized pathophysiological role in β-cell dysfunction in T2DM by repressing insulin synthesis through the STRA6/JAK2/STAT1/ISL-1 signaling pathway, may be selected as a promising therapeutic target for the treatment of β-cell dysfunction in T2DM [19]. In addition, VA exerts its antioxidant function by activating transcriptional networks controlled by retinoic acid receptors (RARs) and retinoic acid X receptors (RXRs) through retinoic acid (RA), which helps diabetic patients reduce the level of free radicals in the body and thus relieve inflammation [59].

The studies shown here appear to suggest that there is an association between the VA level and the development of diabetes, but the results are contradictory for different studies. Whether this phenomenon is associated with a different dietary structure, environment or statistical methods remains to be studied; thus, more studies on the association between VA and diabetes are needed to draw a firm conclusion in the future.

### 3.2. B Vitamins

#### 3.2.1. Biological Functions

B group vitamins, just as the name implies, are a group of compounds. It must be supplemented every day since it can only stay in the body for a few hours as a kind of water-soluble vitamin. As the dispensable nutrients for all human tissues, B vitamins act as coenzymes to participate in the metabolism of sugar, protein and fat. The lack of any B vitamins has negative impacts on the mitochondrial metabolism of amino acids, glucose and fatty acids through the citric acid cycle and electron transport chain [60]. Vitamin B (VB) was once believed to be a single organic compound like vitamin C, but later studies showed that it was actually a group of compounds with different structures named separately, including vitamins B1 (thiamine), B2 (riboflavin), B3 (niacin), B5 (pantothenic acid), B6 (pyridoxine), B12 (cyanocobalamin), B9 (folate) and B7 (biotin) [61]. Although B vitamins are complementary and follow the “cask theory”, they show their own effects [60]. Many of them have been found to be associated with diabetes, but the current evidence is still weak.

#### 3.2.2. B Vitamins and Diabetes

VB1 was discovered as the first vitamin and was isolated in 1926 [62]. One research study on PubMed related to “thiamine” and “diabetes” dated back to 1947; it described thiamine deficiency in diabetic rats as compared to healthy rats [63]. In the past decades, there have been sporadic reports. According to Polizzi et al., people with diabetes and nephropathy exhibited elevated levels of DNA glycation in their leukocytes. However, the levels of DNA glycation decreased after a five-month treatment involving thiamine and pyridoxine supplements [20]. In a cross-sectional investigation, thiamine levels were shown to be lower in people with diabetes compared to healthy controls, diabetes patients with microalbuminuria and diabetes patients with macroalbuminuria. An inverse relationship has also been discovered between thiamine levels and lipid profile in microalbuminuria [21]. Although there was possible selection bias in some of the studies, the results were consistent across the different thiamine markers, suggesting that the requirement of thiamine was higher in individuals with diabetes compared to those without diabetes [64]. Thiamine diphosphate (TDP), the active form of thiamine, may play an important role in this process. TDP, as a coenzyme for transketolase (TK), the pyruvate–dehydrogenase complex and α-ketoglutarate dehydrogenase, plays a fundamental role in intracellular glucose metabolism. In particular, TK is able to shift excess fructose-6-phosphate and glycerhaldeyde-3-phosphate from glycolysis into the pentose phosphate shunt and then eliminate these potentially damaging metabolites from the cytosol [64].

Pyridoxine (B6) is another vitamin that has been reported to be more associated with diabetes. There are six common forms of vitamin B6, namely pyridoxine (PN), pyridoxal (PL), pyridoxamine (PM) and their related 5′-phosphate derivatives (PNP, PLP and PMP) [65]. PLP concentrations are usually measured to assess the level of vitamin B6 in vivo. It has been reported that PLP deficiency could impact diabetes in different ways. For example, Oxenkrug proposed that dysregulation of the tryptophan–kynurenine and kynurenine–nicotinamide adenine dinucleotide metabolic pathways is one of the mechanisms of insulin resistance (IR), while PLP acts as a key enzyme in the kynurenine–nicotinamide pathway, which could be impaired and lead to xanthinic acid over-formation, causing IR when PLP is deficient [22,66]. Another hypothesis is that PLP may impact on insulin resistance by regulating lipid metabolism, and it has been proposed that PLP might activate peroxisome proliferator-activated receptor-γ (PPAR-γ), one of the master nuclear receptors involved in the expression of adipogenesis genes [23]. Finally, there is evidence supporting the idea that PLP deficiency increases homocysteine levels. Elevated homocysteine levels are associated with obesity and impair endothelial function, leading to lipid accumulation in the liver, which, in turn, leads to metabolism abnormalities that form IR [25,67,68]. It is noteworthy that metformin is one of the first-line treatments for T2DM, but the risk of cobalamin deficiency is raised by the long-term use of metformin. Therefore, it is necessary to ensure an adequate amount of vitamin B12 for people with T2DM [69].

In summary, although some studies have reported the association between B vitamins and diabetes, the overall studies are not thorough, and the intervention time of VB is generally short, so there is a lack of convincing evidence. In the future, we still need to design studies with more details to explore the effects of B vitamins on diabetes. Given the complementary effect of B vitamins, the effect of the overall intake of B vitamins on diabetes should also be considered in the daily diet, rather than being limited to a single B-vitamin member.

### 3.3. Antioxidant Vitamins C and E

#### 3.3.1. Biological Functions

Vitamin C (VC, ascorbic acid) is a potent water-soluble antioxidant; it can act as an electron donor that scavenges reactive oxygen species (ROS) and reactive nitrogen species (RNS) to reduce oxidizing agents, and it neutralizes free radicals to protect DNA, proteins and lipids from damage [70]. In addition, it replaces glucose in many biochemical reactions due to their structural similarity and prevents non-enzymatic glycosylation [71]. Vitamin E (VE) refers to a family of fat-soluble compounds that includes tocopherol and tocotrienol, also known as α-tocopherol. VE, as a putative radical scavenger, is probably the most important inhibitor of radical-induced lipoprotein lipid peroxidation [72]. This lipid-soluble agent can readily cross cell membranes and exert its effects both intracellularly and in membranes. In addition, it can extinguish single oxygen species, as well as terminate free radical chain reactions [29]. Oxidative stress is a state of imbalance between oxidation and antioxidation in the body. Studies have found that markers of oxidative stress are elevated in patients with diabetes and have confirmed that oxidative stress can stimulate insulin secretion and glucose metabolism [70,73,74,75,76]. As our understanding of diabetes has grown, it has become evident that oxidative and nitrosative redox alterations are underlying factors in the pathogenesis of diabetes and their associated health complications [77]. Consequently, antioxidant supplements have been proposed as one of the means of diabetes treatment. Undoubtedly, VC and VE, as well-known antioxidants, have also become the focus of diabetes research [29].

#### 3.3.2. Vitamins C and E and Diabetes

The levels of blood glucose and HbA1C are a central measure of risk of diabetes and its complications [28]. According to a meta-analysis that collected data from 1980 to 2020, although there is no adequate evidence to support VC supplementation for dyslipidemias in diabetic patients, VC supplementation can reduce the levels of FBS and HbA1C, especially in younger diabetic patients [26]. Over the past 5 years, at least three similar meta-analyses have demonstrated that VC supplementation effectively reduced the levels of fasting glucose and HbA1C in patients [27,28,78]. Similarly, VE has also been found to be effective in the management of T2DM. Several randomized controlled trials provided evidence that the effects of VC and VE can restore insulin, lipid profile, fasting blood sugar (FBS), homeostasis model assessment of insulin resistance (HOMA-IR), reduced glutathione (GSH) and Quantitative Insulin Sensitivity Check Index (QISCI) to novel values. Moreover, the author suggested that these possible protective effects of VC and VE may be mediated through increasing QISCI and improving the non-enzymatic antioxidant system [29,30,31,79]. Fox example, the studies observed deceases in plasma concentrations of ROS and oxidative stress with an increase in VC concentration, concomitant with improved insulin sensitivity, after VC supplementation in patients with T2DM [32,80]. In addition, Rhee et al. found that superoxide radical concentrations in the liver of streptozotocin-induced diabetic rats were significantly higher than those of control livers. VE supplementation reduced liver superoxide radical concentrations in a dose-dependent manner [33]. Changes in redox signaling promote the attenuated activation of stress signaling pathways (including NF-κB, JNK and MAPK), and this finding has been verified by in vitro and animal studies [33,81,82]. Overall, the antioxidant properties of VC and VE may improve insulin sensitivity by altering oxidative stress signaling, but more clinical trials are needed to confirm this evidence.

On the one hand, it has been reported that VC and VE can control the symptoms of hyperglycemia in diabetic patients by reducing lipid peroxidation and decreasing the glycation of insulin in the pancreas, thus indicating that the ways that antioxidant VC and VE supplements can improve the symptoms of diabetes were complex and multi-directional [35,36]. Significantly, VC is one of the effective reducing agents in the body which plays a key role in scavenging free radicals and protecting and maintaining the reduction of some important substances such as VE and GSH [83]. For instance, VC reduces tocopherol radical formed during lipid peroxidation back to α-tocopherol and, thus, may protect against lipid peroxidation in part by regenerating alpha tocopherol. It was found that VC-deficient rats have been found to have decreased levels of VE in their tissue, along with increased lipid peroxidation, suggesting that VC-reduction protection of VE has been working in the body [84]. Therefore, the interaction between VC and VE probably serves as an important network of redox regulation within body cells and tissues.

It should be emphasized that antioxidant treatment has been widely shown to be effective in reducing oxidative stress and improving insulin resistance and glycemic outcomes in rodent models and in cell-based experiments. However, the study findings showed that antioxidant treatments in humans were not always effective, and, in some cases, they even showed adverse effects. The reason for this may be related to the subjects: a meta-analysis of studies examining the effects of VC administration on glucose, HbA1c and insulin concentrations selected in the databases PubMed, Embase, Scopus and Cochrane Library showed that patients with diabetes and older participants, as well as studies with longer durations, had relatively greater reductions in glucose levels [37].

Beyond that, it is affected by the baseline vitamin levels of the subjects. We found that VC-deficient patients showed significantly greater improvement in HbA1C and greater reduction in plasma oxidative stress after VC supplementation compared with participants with normal VC status [27]. Finally, the dose of vitamin supplementation must be considered. While most studies recommend discreetly supplementing with 500–1000 mg of VC per day, individuals with β-thalassemia and/or end-stage renal disease should aim for lower levels of intake under medical supervision to avoid potential harm [85]. On the other hand, infections or sepsis common in diabetics may require a greater VC intake to achieve and maintain optimal concentrations [86,87]. Therefore, we are more in favor of the rational choice of antioxidant vitamin supplements for personalized treatment of patients with diabetes according to their physical conditions, rather than blindly supplementing with mythical VC and VE products.

### 3.4. Vitamin D

#### 3.4.1. Biological Functions

Over the past 100 years, many major breakthroughs and discoveries have occurred in relation to vitamin D (VD). For a long time, VD has been mistaken for VA to prevent the symptoms of rickets. It was not until 1922 that McLean et al. conducted a set of dietary experiments and realized that it was in fact a new vitamin, which has been called the “fourth vitamin”, that is, vitamin D [88]. After 1930, the chemical structure of VD was determined, and the chemical properties of VD2 and VD3 were clarified in 1932 and 1936, respectively [89]. In 1972, 1,25(OH)_2_D, the active metabolic form of vitamin D, was discovered and found to be effective in preventing osteoporosis by promoting the absorption and circulation of calcium and phosphorus through three pathways [90,91,92].

VD’s development has caused qualitative changes in the last 20 years. People are no longer limited to its role in promoting the absorption of calcium and phosphorus; they have gradually recognized that VD is a hormonogen, and its receptor (VDR) is widely distributed throughout the brain, heart, pancreas, B lymphocytes and other tissues. Deficiency of vitamin D may cause many diseases, including hypertension, colon cancer and diabetes [93,94,95]. Thus, the use of VD supplements has been proposed as a potential intervention for reducing diabetes risk.

Vitamin D is usually obtained from food, but few natural foods contain enough of VD; cod liver oil and oily fish are considered to be rich sources, while easily available milk or breast milk contains only trace amounts of VD [96]. Most VD, in fact, comes from our own skin, with which we convert 7-dihydrocholesterol into pre-VD that is subsequently transported to the liver. Here, VD is hydroxylated by liver 25-hydroxylase (CYP2R1). The resulting 25(OH)D3 is then hydroxylated in the kidney by 1-α-hydroxylase (CYP27B1), generating the active hormone 1,25(OH)_2_D [97].

#### 3.4.2. Vitamin D and Diabetes

VD insufficiency has long been suspected to be a risk factor for diabetes, and this theory has been supported in some observational studies. It has been reported that there were significantly more children with diabetes born during the spring–summer than in autumn–winter in Greece during the period 1978–2008 [38]. Similarly, seasonal variation was also observed with cases increasing during the cold months in Germany [98], Denmark [39] and China [40]. In addition, statistics found that Sardinia and Finland are the two regions with the highest incidence of T1D in the world, and there are also seasonal patterns [99]. The researchers believed that latitude and solar radiation showed influence in the incidence of TD, and VD was the first factor affected by both elements. It was also confirmed by the World Health Organization Diabetes Mondiale, which analyzed the data of children aged 0–14 years between 1990 and 1999 and found that seasonal variation in T1D incidence rates in children under 15 years of age was a real phenomenon [100]. However, these are only hypotheses based on observation; other factors, such as sedentary behaviors, winter colds, lifestyle changes, etc., may also be contributing to this outcome. In light of this, genetic studies have also searched for a link between TD and the VD system. The United Kingdom reported that genetic variations in CYP27B1, a key enzyme involved in VD metabolic enzymes, affects susceptibility to T1D [101]. In addition, DHCR7 and CYP2R1 have also been found to be linked to T1D [102], whereas the largest study to date found no association between several VDR-gene single nucleotide polymorphisms (SNPs) and T1D [103]. Thus, epidemiology points towards a role for the VD system in the onset of TD, but the genetic data are conflicting. In order to verify this, more controlled intervention experiments have been conducted. Determining how to prevent β-cell exhaustion under diabetic conditions is a major therapeutic challenge. Wei et al. used a compound named IBRD9 in combination with VD to treat T2DM mice and found that IBRD appeared to increase the activation of VD receptors, thereby improving the survival of be β-cells, and this combination treatment has been shown to return blood sugar to normal levels in diabetic mice [41]. In addition, Mendes et al. investigated the short-term effect of 1α,25(OH)_2_D_3_ and cholecalciferol on the glycemia and insulin sensitivity of control and dexamethasone-induced insulin-resistance rats, and the results showed that 1α,25(OH)_2_D_3_ (short-term effect) plays an unprecedented role in regulating glucose homeostasis and preventing insulin resistance [42]. The effectiveness of animal studies has also led to interest in clinical research. Panjiyar et al. treated 42 children aged 6–12 years with T1D, using 3000IU of cholecalciferol daily. With another 30 patients as a case control group, they found a significantly smaller decline in C-peptide in the D-treated group and a reduction in HbA1c levels [43]. Similarly, the results of a clinical study in an Indian hospital concluded that oral VD may serve as an adjuvant to insulin therapy for children with T1DM by augmenting residual beta-cell function and improving insulin secretion [44]. Recently, a meta-analysis that included 4190 subjects in three studies evaluated whether the administration of VD decreases the risk for diabetes among people with prediabetes and concluded that VD reduced the risk for diabetes by 15% and the absolute sick reduction of 3.3% [104]. Interestingly, a randomized double-blind clinical trial in the United States had the opposite result. Pittas et al. screened people at high risk of prediabetes for daily VD supplementation and found that a daily VD3 supplement of 4000IU did not significantly reduce the risk of diabetes in people at high risk without VD deficiency [45]. Obviously, current experimental studies cannot fully explain these contradictory results. According to the current known pathogenesis of diabetes, T1D is caused by the failure of pancreatic β-cells to secrete normally due to the destruction of the immune system. T2D results from impaired β-cell function, increased insulin resistance and systemic inflammation, and VD may influence the pathways all of above.

Mechanistically, 1,25(OH)_2_D can bind directly to VDR in β-cells, and 1,25(OH)_2_D directly stimulates the expression of the insulin receptor and promotes insulin secretion [45,105]. A large number of data support the claim that VD deficiency in rats can lead to impaired insulin secretion and glucose tolerance, and VD supplementation can increase insulin secretion by 48% to improve the damage [46]. In line with this, the laboratory of Christina Cade also demonstrated that 1,25(OH)_2_D_3_ improved impaired glucose clearance and insulin secretion in VD-deficient rats; a dose dependence of this hypoglycemic action was also established [106]. In some clinical studies, VD supplementation has also been found to significantly increase peripheral insulin sensitivity and β-cell function, suggesting that it may slow metabolic deterioration in diabetic patients [47,107]. In addition, an important part of the beneficial effect of VD on TD is the protection of the immune system. It has been found that almost all immune cells express VDR, including monocytes, macrophages and neutrophils [108,109,110]. At the same time, 1,25(OH)_2_D_3_ combined with VDR can downregulate the activity of NF-κB and reduce the expression of IL-1, IL-6 and TNF-α in monocytes, while inflammation impairs β-cell function and insulin sensitivity [111]. However, caution is still needed to interpretate the results of VD intervention in in vivo studies. The symptoms of diabetes not only include VD deficiency but also altered parathyroid hormone (PTH) levels, altered caloric intake and hypocalcemia. Hypocalcemia, in particular, has been shown to dramatically alters β-cell function. Moreover, VD is a growth factor that promotes calcium absorption, making it difficult to determine whether DM may be caused by VD deficiency or be a direct result of calcium deficiency [111].

To sum up, most animal studies showed that VD deficiency increases the risk of TD and conversely that treatment with VD decreases the risk. However, in the long run, it cannot be ruled out that this may be due to the immunological effect of VD deficiency. It is difficult to draw definitive conclusions from conflicting clinical trial results, but one statement concerning VD and diabetes can be made with a reasonable degree of certainty: severe VD deficiency should be avoided. VD supplementation may not be therapeutic after the onset of the disease, but it still has a high possibility of delaying the risk of diabetes in high-risk people.

### 3.5. Vitamin K

#### 3.5.1. Biological Functions

Vitamin K is a fat-soluble vitamin with two natural forms: VK1 (phylloquinone) and VK2 (menaquinones), which represent a family of fat-soluble compounds sharing a common chemical structure of 2-methyl-1,4-napthoquinone [112]. VK1 is widely found in leafy greens and algae, while VK2 can be produced by an array of bacteria, such as gut microbes or bacteria capable of carrying out fermentation [113]. As a result, VK deficiency is less common in adults, while it is usually found in infants since the amount of phylloquinone obtained from breast milk is limited. As is well known, VK plays an important role in regulating blood homeostasis and bone metabolism, such as osteoporosis, cardiovascular disease and rheumatoid arthritis; these diseases all share a common feature of chronic low-grade inflammation and oxidative stress, thus suggesting that VK is largely involved in the expression of genes associated with oxidative stress and inflammatory response [114]. This hypothesis was confirmed in animal studies. Ohsaki et al. showed that VK inhibits LPS-induced inflammation in rats and macrophages by mediating the inactivation of the NF-κB signaling pathway [55]. Furthermore, it has been reported that VKH2 (the reduced form of VK) was able to prevent a redox imbalance by decreasing ROS levels, which may be the role of VK as an antioxidant inflammation [115].

#### 3.5.2. Vitamin K and Diabetes

Several different types of studies have reported an association between VK and diabetes, including observational studies, clinical intervention studies and Mendelian randomization studies. In a cross-sectional study assessing the association between the Mediterranean Diet and markers of metabolic risk associated with T2DM, the PREDIMED center in Spain showed for the first time that increasing dietary intake of phylloquinone can improve insulin resistance, supporting the protective role of VK1 in low-grade chronic inflammatory disease [48]. Similarly, Yoshida et al. selected 2719 people without diabetes in the Framingham Offspring study to evaluate the association between phylloquinone intake, insulin sensitivity and blood glucose status, and their founding suggested that phylloquinone intake may have a beneficial effect on glucose homeostasis, or it may serve as a surrogate marker of other dietary or lifestyle factors that were not controlled in this analysis [49]. In addition, Dihingia et al. conducted a case–control experiment in India comparing VK1 levels in 25 patients with T2DM and 20 healthy volunteers and found that the average fasting plasma VK1 level in diabetic patients (1.93 nmol/L) was lower than that in the normal population (5.28 nmol/L). Moreover, fasting blood glucose was negatively correlated with plasma VK1 concentration in diabetic patients [116]. The European Prospective Study of Cancer and Nutrition in the Netherlands, which included 38,094 subjects and followed up for up to 10 years, showed that the risk of diabetes decreased with the increase in VK1 and VK2 intake [50]. Similar results were also reported in the Iranian IMOS research center and the Spanish PREDIMED research center [48,117].

At present, most clinical studies exploring the intervention of VK in diabetes mainly focus on diabetes-related metabolic indicators. For example, Sakamoto et al. conducted a non-randomized clinical intervention study in Japan. Twelve young healthy men were divided into three groups according to baseline serum descarboxy prothrombin (DP) levels, and blood glucose status indexes were similar in all groups before intervention. After 1 week of VK2 supplementation, the level of immunoreactive insulin (IRI) at 2 h postprandial in the group with the highest DP level at baseline was significantly lower than that before intervention (*p* < 0.05). These results suggested that, in subclinical VK-deficient patients with high serum DP levels, appropriate supplementation of VK can regulate insulin metabolism, and the increase in VK intake is associated with the decrease in IRI [51]. In addition, Choi et al. randomly divided 42 healthy Korean men into a VK2 supplementation group and control group. After 4 weeks of intervention, the insulin sensitivity index of the experimental group was significantly higher than that at baseline, and the glucose disposal index was also significantly higher than that before the intervention, while there was no significant change in the control group. This suggested that VK2 may have a role in enhancing insulin sensitivity in healthy men [52]. Mendelian randomization (MR) is a type of study that uses genetic variation to infer causality between exposure and disease outcomes. Zwakenberg et al. used MR analysis data from three studies: the European Prospective Study of Cancer and Nutrition, the Diabetes Genetics Replication and Meta-analysis, and the UK Biobank. The causal relationship between blood VK1 concentration and T2DM mellitus was investigated. A total of 69,647 patients with T2DM and 551,336 participants without diabetes were included in the study; the researchers calculated weighted genetic risk scores, and the inverse variance weighted analysis found that each natural log-transformed unit (nmol/L) increase in serum VK1 was associated with a 7% reduction in the risk of T2DM. Therefore, this study also further suggested that the circulating VK1 concentration may be associated with the reduced risk of T2DM at the genetic level [53].

At present, the mechanism of the effect of VK on diabetes is not clear, while the existing studies suggested that it may be achieved by improving insulin resistance and thus affecting the body’s glucose metabolism in the following three ways. One is the regulation of osteocalcin (OC). OC is a VK-dependent protein that has been shown in animal studies to promote β-cell proliferation and insulin secretion, as well as increase insulin sensitivity [54,118]. In addition, clinical studies also support that OC plays an important role in glucose metabolism by increasing insulin secretion and adiponectin expression [119,120,121]. The second way is through an anti-inflammatory effect; it has been established that inflammation can induce insulin resistance. Ohsaki et al. found that VK can inhibit the activation of NF-κB signaling pathway by inhibiting IKKα/β phosphorylation, so it can reduce the expression of inflammatory factors and help improve insulin resistance [55]. The third way is the lipid-lowering effect. Lipid accumulation in obese patients can cause a decrease in insulin sensitivity [122]. Sogabe et al. found that the long-term intake of VK2 in SD rats significantly reduced total fat mass and serum glyceride levels, thereby alleviating insulin resistance [56].

According to the above studies, the potential effect of VK on diabetes has been preliminarily confirmed, but most of these studies use food frequency questionnaire (FFQ) to collect and analyze the nutritional status of VK in individuals, but according to the test of Beulens et al., it is suggested that the use of FFQ will bring large systematic error and random error [50]. Beyond that, OC and matrix Gla protein (MGP) are commonly used as VK biomarkers, but whether these biomarkers can be used as reliable criteria to assess VK exposure or effect is still unclear [123]. Therefore, some studies have considered that VK is not significantly associated with diabetes. In conclusion, it is still unclear whether VK deficiency is the cause or result of diabetes. In the future, multicenter, multi-ethnic, large-sample prospective cohort studies and clinical intervention studies are needed to verify the relationship between VK deficiency and diabetes.

## 4. Conclusions

This review collected studies on the relationship between essential micronutrients and diabetes mellitus and summarized the possible mechanisms. This review showed that essential micronutrients may affect the development of diabetes through various pathways, such as antioxidation and reducing islet sensitivity. However, it should not be ignored that some studies believed that there was no significant association between essential micronutrients and diabetes. It may be caused by the inconsistency in the inclusion criteria for studies, the short duration of micronutrients intervention and the relatively concentrated ethnicity of the subjects in a single study. Therefore, although there are indications that micronutrients might beneficially regulate glucose and lipid metabolism, the available evidence is still inconclusive. Thus, we do not recommend it as a routine treatment for diabetes at this time. According to the investigation results, we found that the use of vitamin supplements in developed countries is rising dramatically, yet most users are unaware of the potential risks of overdose and drug interactions. At the same time, in some developing and poor countries, the public is suffering from a range of diseases caused by vitamin deficiency. In view of the importance of micronutrients for human health, we believe that it is necessary to improve the public’s awareness of micronutrients and recommend that the public should adjust the dietary structure in their daily diet to ensure the intake of essential micronutrients according to their own health status.

In the future, larger, better and multi-ethnic prospective cohort studies and clinical intervention studies should be performed, with the help of tools such as MR, metagenomics and machine learning to mine and analyze experimental data in order to elucidate the causal relationship between vitamins and diabetes.

## Figures and Tables

**Table 1:** Summary of the association between different types of vitamins and diabetes.

Reference	Study Type	Subjects	Conclusion (Key Finding)
Vitamin A			
[10]	Cohort study	Postmenopausal women, aged 50–79 y, *n* = 153	Lower risks of diabetes were associated with a higher intake of α- and β-carotene.
[11]	Prospective study	Human, *n* = 37,846	Carotenoids are known to have antioxidant functions, which may underlie the observed inverse associations with diabetes.
[12]	Randomized controlled trial	Male smokers aged 50–69 y, *n* = 29,133	Supplementation with α-tocopherol or β-carotene had no preventive effect on the risk of type 2 diabetes in middle-aged male smokers.
[13]	Prospective study	Adults without cardiovascular disease, aged 45–84 y, *n* = 6814	β-carotene was not associated with the risk of T2D.
[14]	Prospective cohort study	Healthy Japanese, aged 40–79 y, *n* = 19,168	A higher dietary intake of fat-soluble vitamins K and E, but not vitamin A or D, was associated with lowered risk of type 2 diabetes among the Japanese population.
[15]	Prospective cohort study	Adults (8537 men and 8577 women), *n* = 17,111	The adequate intake of vitamin A may help protect against diabetes, especially in men.
[16]	Animal experiment	Dietary VA deficiency model mice	VA deficiency induced pancreatic islets dysfunction by activating the ISC population.
[17]	Animal experiment	C57BL/6, LRAT−/− (lecithin retinol acyltransferase null)	VA is essential for the maintenance of β-cell functions in adult pancreas.
[18]	Prospective study	Prediabetes, *n* = 1011	A U-shaped relationship existed between serum retinol-binding protein 4 levels (>55 μg/mL or <31 μg/mL) and the risk of incident type 2 diabetes in subjects with prediabetes.
[19]	Animal experiment	Transgenic mice expressing human RBP4	Retinol-binding protein 4 represses insulin synthesis through the STRA6/JAK2/STAT1/ISL-1 signaling pathway.
Vitamin B			
[20]	Randomized controlled trial	Patients with diabetes, *n* = 31	The combined administration of vitamins B1 and B6 to diabetic nephropathy patients causes a decrease in DNA glycation in leukocytes.
[21]	Cross-sectional study	Microalbuminurics type 2 diabetics (*n* = 20), healthy individuals, *n* = 20, macroalbuminuric type 2 diabetics, *n* = 20	Thiamine levels were reduced in the diabetic population, and this reduction in thiamine levels was negatively correlated with the lipid profile in microalbuminuric diabetics.
[22]	Animal experiment	Vitamin B6-deficient rats	Vitamin B6 supplementation could normalize urinary 3-hydroxykynurenine and xanthurenic levels in rats.
[23]	Cross-sectional studies, animal experiment, cell experiments	Participants (BMI 20–68 kg/m^2^); 3T3-L1, SAT and VAT adipocyte cells	These results support the notion that the in situ production of PLP is required for physiological adipogenesis.
[24]	Cell experiments	3T3-L1 adipocyte cells	Vitamin B6 can act as an activator for PPARγ, which may contribute to the antitumor and anti-inflammatory effects of vitamin B6.
[25]	Animal experiment	Mice with high-fat diet (HFD)	Vitamin B6 protects endothelial function and improves insulin resistance, and low Vitamin B6 status might be a risk factor for NAFLD.
Vitamins C and E			
[26]	Meta-analysis	Type 2 diabetic patients and vitamin C as key words	There is no adequate evidence to support vitamin C supplementation for dyslipidemias in diabetic patients. Specific groups of patients might have benefited, including younger diabetic patients.
[27]	Meta-analysis	Randomized controlled trials related to diabetes	Vitamin C supplementation may be potentially effective for improving glycemic control and BP in people with type 2 diabetes.
[28]	Meta-analysis	Randomized controlled trials related to diabetes	The administration of chromium, CoQ10, vitamin C and vitamin E as add-on supplements for patients with T2DM resulted in significant effects on important glycemic control parameters.
[29]	Randomized controlled trial	T2DM male patients, *n* = 40	Antioxidant vitamin supplementation (VC/VE) can improve the clinical status of type 2 diabetes mellitus and reduce or prevent the pathogenesis and complications of diabetes.
[30]	Randomized controlled trial	Type 2 diabetic patients, *n* = 50	Vitamin C may have beneficial effects on HDL-C in diabetic patients, without having significant effects on plasma glucose or other lipid parameters.
[31]	Randomized controlled trial	Patients with T2DM mellitus, *n* = 70	Vitamin C is a powerful adjunct to the treatment of T2DM.
[32]	Randomized controlled trial	T2DM patients, *n* = 40	Chronic vitamin C administration has beneficial effects upon glucose and lipid metabolism in aged non-insulin-dependent (type II) diabetic patients.
[33]	Animal experiment	Diabetic rats	Vitamin E can regulate the activity of phospholipase A(2) and PLA(2), reduce the production of reactive oxygen species and destructive oxides and maintain the fluidity of liver cell membrane in diabetic rats.
[34]	Animal experiment	Male Wistar rats	Antioxidants (VC) reduce insulin resistance induced by obesity/dyslipidemia in humans.
[35]	Animal experiment	OB/OB mice	Vitamin C supplementation can decrease insulin glycation and ameliorate aspects of the obesity–diabetes syndrome in ob/ob mice.
[36]	Randomized controlled trial	Participants with least three features of metabolic syndrome, *n* = 80	Combined supplementation of α-tocopherol AT + γ-tocopherol GT was able to reduce oxidative and nitration stress and inflammation levels in subjects with metabolic syndrome.
[37]	Meta-analysis	Vitamin C + glucose + insulin + HbA1c	Age, baseline BMI, plasma glucose levels and effect size were the modifiers of the effect of vitamin C on insulin concentration.
Vitamin D			
[38]	Retrospective study	Children with T1DM, *n* = 1148	The study supports the concept of seasonality in T1DM diagnosis, implying a possible relationship between clinical expression of T1DM and various climatic factors.
[39]	Retrospective study	Children with diabetes, *n* = 2166	There is a significant seasonal variation in the incidence of T1DM in Danish children, which may be related to virus prevalence, sun exposure or vitamin D levels.
[40]	Prospective study	Pregnant women, *n* = 2658	The polymorphisms of the VD metabolic pathway gene were associated with gestational 25(OH)D, and the associations differ by seasons and VD supplements.
[41]	Cell experiment; Animal experiment	Db/db mice; β cell	Activation of VDR combined with the dismissal of the BAF complex is able to improve β-cell function and thereby glucose homeostasis in an inflammation-driven diabetes model.
[42]	Animal experiment	Insulin resistance rat model	This is the first study highlighting the unprecedented role of 1,25-D_3_ (short-term effect) in the regulation of glucose homeostasis and on the prevention of insulin resistance.
[43]	Randomized controlled trial	Children with T1D, *n* = 42	Sustained serum 25-(OH)D concentrations with cholecalciferol supplementation for one year improves metabolic control and slows the decline of RBCF in children with T1DM.
[44]	Randomized controlled trial	Children with T1D, *n* = 52	Oral vitamin D may serve as an adjuvant to insulin therapy for children with T1DM by augmenting residual beta-cell function and improving insulin secretion.
[45]	Randomized controlled trial	Adults with prediabetes, *n* = 2423	Among persons at high risk for T2DM not selected for vitamin D insufficiency, vitamin D3 supplementation at a dose of 4000 IU per day did not result in a significantly lower risk of diabetes than the placebo.
[46]	Animal experiment	Vitamin D deficiency rats	Pancreases from vitamin D-deficient rats exhibited a 48 percent reduction in insulin secretion compared to that for pancreases from vitamin D-deficient rats that had been replenished with vitamin D.
[47]	Randomized controlled trial	Participants with T2DM, *n* = 96	Vitamin D supplementation significantly improves peripheral insulin sensitivity and β-cell function, suggesting that it may slow metabolic deterioration in this population.
Vitamin K			
[48]	Cross-sectional analysis	Men (aged 55–80), women (aged 60–80 years), *n* = 568	Increasing dietary phylloquinone intake reduces inflammation and inflammation-related molecules.
[49]	Cross-sectional analysis	Human, aged 26–81, *n* = 2719	Phylloquinone has beneficial effects on glucose homeostasis in both men and women.
[50]	Prospective cohort study	Human, aged 20–70 y, *n* = 38,094	Phylloquinone and menaquinones intakes may be associated with a reduced risk of T2DM.
[51]	Randomized controlled trial	Man, aged 21, *n* = 12	Appropriate supplementation of vitamin K can regulate insulin metabolism, and the increase in vitamin K intake is associated with the decrease in immunoreactive insulin.
[52]	Randomized controlled trial	Healthy man, *n* = 42	Vitamin K2 may have a role in enhancing insulin sensitivity in healthy men.
[53]	Mendelian randomization	Patients with T2DM, *n* = 69,647, participants without diabetes, *n* = 51,336	The circulating VK1 concentration may be associated with the reduced risk of T2DM at the genetic level.
[54]	Animal experiment	C57BL/6J, fill with osteocalcin	Osteocalcin could promote β-cell proliferation and insulin secretion, as well as increase insulin sensitivity.
[55]	Cell experiment	Human monocytic THP-1 and mouse RAW264.7 cells	The anti-inflammatory activity of vitamin K is mediated via the inactivation of the NFκB signaling pathway.
[56]	Animal experiment	Sprague Dawley rats	Long-term intake of vitamin K2 in SD rats significantly reduced total fat mass and serum glyceride levels, thereby alleviating insulin resistance.

## Data Availability

Not applicable.

## References

[1] Demir S., Nawroth P.P., Herzig S., Ekim Üstünel B. (2021). Emerging Targets in Type 2 Diabetes and Diabetic Complications. Adv. Sci. (Weinh).

[2] Adeghate S.E. (2017). Chronic Complications of Diabetes Mellitus: A Mini Review. Curr. Diabetes Rev..

[3] Sun H., Saeedi P., Karuranga S., Pinkepank M., Ogurtsova K., Duncan B.B., Stein C., Basit A., Chan J.C.N., Mbanya J.C. (2022). IDF Diabetes Atlas: Global, Regional and Country-Level Diabetes Prevalence Estimates for 2021 and Projections for 2045. Diabetes Res. Clin. Pract..

[4] American Diabetes Association Professional Practice Committee (2021). 2. Classification and Diagnosis of Diabetes: Standards of Medical Care in Diabetes—2022. Diabetes Care.

[5] Kerru N., Singh-Pillay A., Awolade P., Singh P. (2018). Current Anti-Diabetic Agents and Their Molecular Targets: A Review. Eur. J. Med. Chem. Chim. Ther..

[6] Kayaniyil S., Vieth R., Retnakaran R., Knight J.A., Qi Y., Gerstein H.C., Perkins B.A., Harris S.B., Zinman B., Hanley A.J. (2010). Association of Vitamin D with Insulin Resistance and β-Cell Dysfunction in Subjects at Risk for Type 2 Diabetes. Diabetes Care.

[7] Palomer X., Gonzalez-Clemente J.M., Blanco-Vaca F., Mauricio D. (2008). Role of Vitamin D in the Pathogenesis of Type 2 Diabetes Mellitus. Diabetes Obes. Metab..

[8] Rösen P., Nawroth P.P., King G., Möller W., Tritschler H.J., Packer L. (2010). The Role of Oxidative Stress in the Onset and Progression of Diabetes and Its Complications: A Summary of a Congress Series Sponsored by UNESCO-MCBN, the American Diabetes Association and the German Diabetes Society. Diabetes Metab. Res. Rev..

[9] Blaner W.S. (2019). Vitamin A Signaling and Homeostasis in Obesity, Diabetes, and Metabolic Disorders. Pharmacol. Ther..

[10] Prentice R.L., Pettinger M., Neuhouser M.L., Tinker L.F., Huang Y., Zheng C., Manson J.E., Mossavar-Rahmani Y., Anderson G.L., Lampe J.W. (2019). Application of Blood Concentration Biomarkers in Nutritional Epidemiology: Example of Carotenoid and Tocopherol Intake in Relation to Chronic Disease Risk. Am. J. Clin. Nutr..

[11] Sluijs I., Cadier E., Beulens J.W., van der A. D.L., Spijkerman A.M., van der Schouw Y.T. (2015). Dietary intake of carotenoids and risk of type 2 diabetes. Nutr. Metab. Cardiovasc. Dis..

[12] Kataja-Tuomola M., Sundell J.R., Männistö S., Virtanen M.J., Kontto J., Albanes D., Virtamo J. (2008). Effect of Alpha-Tocopherol and Beta-Carotene Supplementation on the Incidence of Type 2 Diabetes. Diabetologia.

[13] de Oliveira Otto M.C., Alonso A., Lee D.H., Delclos G.L., Bertoni A.G., Jiang R., Lima J.A., Symanski E., Jacobs D.R., Nettleton J.A. (2012). Dietary Intakes of Zinc and Heme Iron from Red Meat, But Not from Other Sources, Are Associated with Greater Risk of Metabolic Syndrome and Cardiovascular Disease. J. Nutr..

[14] Eshak E.S., Iso H., Muraki I., Tamakoshi A. (2019). Fat-Soluble Vitamins from Diet in Relation to Risk of Type 2 Diabetes Mellitus in Japanese Population. Br. J. Nutr..

[15] Su L., He J., Liu Z., Wu S., Chen P., Li K., Fang A. (2022). Dietary Total Vitamin A, β-Carotene, and Retinol Intake and the Risk of Diabetes in Chinese Adults with Plant-Based Diets. J. Clin. Endocrinol. Metab..

[16] Zhou Y., Zhou J., Sun B., Xu W., Sun Z. (2020). Vitamin A Deficiency Causes Islet Dysfunction by Inducing Islet Stellate Cell Activation via Cellular Retinol Binding Protein 1. Int. J. Biol. Sci..

[17] Trasino S.E., Benoit Y.D., Gudas L.J. (2015). Vitamin A Deficiency Causes Hyperglycemia and Loss of Pancreatic β-Cell Mass. J. Biol. Chem..

[18] Fan J., Yin S., Lin D., Liu Y., Xia M. (2019). Association of Serum Retinol-Binding Protein 4 Levels and the Risk of Incident Type 2 Diabetes in Subjects with Prediabetes. Diabetes Care.

[19] Huang R., Bai X., Li X., Wang X., Zhao L. (2020). Retinol-Binding Protein 4 Activates STRA6 Provoking Pancreatic β Cell Dysfunction in Type 2 Diabetes. Diabetes.

[20] Polizzi F.C., Andican G., Çetin E., Civelek S., Yumuk V., Burçak G. (2012). Increased DNA-Glycation in Type 2 Diabetic Patients: The Effect of Thiamine and Pyridoxine Therapy. Exp. Clin. Endocrinol. Diabetes.

[21] Waheed P., Naveed A.K., Ahmed T. (2013). Thiamine Deficiency and Its Correlation with Dyslipidaemia in Diabetics with Microalbuminuria. J. Pak. Med. Assoc..

[22] Takeuchi F., Tsubouchi R., Izuta S., Shibata Y. (1989). Kynurenine metabolism and Xanthurenic Acid Formation in Vitamin B6-Deficient Rat after Tryptophan Injection. J. Nutr. Sci. Vitaminol..

[23] Moreno-Navarrete J.M., Jove M., Ortega F., Xifra G., Ricart W., Obis È., Pamplona R., Portero-Otin M., Fernández-Real J.M. (2016). Metabolomics Uncovers the Role of Adipose Tissue PDXK in Adipogenesis and Systemic Insulin Sensitivity. Diabetologia.

[24] Yanaka N., Kanda M., Toya K., Suehiro H., Kato N. (2011). Vitamin B6 Regulates mRNA Expression of Peroxisome Proliferator-Activated Receptor-γ Target Genes. Exp. Ther. Med..

[25] Liu Z., Li P., Zhao Z.H., Zhang Y., Ma Z.M., Wang S.X. (2016). Vitamin B6 Prevents Endothelial Dysfunction, Insulin Resistance, and Hepatic Lipid Accumulation in *Apoe*(−/−) Mice Fed with High-Fat Diet. J. Diabetes Res..

[26] Tareke A.A., Hadgu A.A. (2021). The Effect of Vitamin C Supplementation on Lipid Profile of Type 2 Diabetic Patients: A Systematic Review and Meta-Analysis of Clinical Trials. Diabetol. Metab. Syndr..

[27] Mason S.A., Keske M.A., Wadley G.D. (2021). Effects of Vitamin C Supplementation on Glycemic Control and Cardiovascular Risk Factors in People with Type 2 Diabetes: A GRADE-Assessed Systematic Review and Meta-Analysis of Randomized Controlled Trials. Diabetes Care.

[28] Kim Y., Oh Y.K., Lee J., Kim E. (2022). Could Nutrient Supplements Provide Additional Glycemic Control in Diabetes Management? A Systematic Review and Meta-Analysis of Randomized Controlled Trials of as an Add-on Nutritional Supplementation Therapy. Arch. Pharm. Res..

[29] El-Aal A.A., El-Ghffar E.A.A., Ghali A.A., Zughbur M.R., Sirdah M.M. (2018). The Effect of Vitamin C and/or E Supplementations on Type 2 Diabetic Adult Males under Metformin Treatment: A Single-Blinded Randomized Controlled Clinical Trial. Diabetes Metab. Syndr. Clin. Res. Rev..

[30] Siavash M., Amini M. (2014). Vitamin C May Have Similar Beneficial Effects to Gemfibrozil on Serum High-Density Lipoprotein-Cholesterol in Type 2 Diabetic Patients. J. Res. Pharm. Pract..

[31] Dakhale G.N., Chaudhari H.V., Shrivastava M. (2011). Supplementation of Vitamin C Reduces Blood Glucose and Improves Glycosylated Hemoglobin in Type 2 Diabetes Mellitus: A Randomized, Double-Blind Study. Adv. Pharmacol. Sci..

[32] Paolisso G., Balbi V., Volpe C., Varricchio G., Gambardella A., Saccomanno F., Ammendola S., Varricchio M., D’Onofrio F. (1995). Metabolic Benefits Deriving from Chronic Vitamin C Supplementation in Aged Non-Insulin Dependent Diabetics. J. Am. Coll. Nutr..

[33] Rhee S.J., Jeong Y.C., Choi J.H. (2005). Effects of Vitamin E on Phospholipase A2 Activity and Oxidative Damage to the Liver in Streptozotocin-Induced Diabetic Rats. Ann. Nutr. Metab..

[34] Vinayagamoorthi R., Bobby Z., Sridhar M.G. (2008). Antioxidants Preserve Redox Balance and Inhibit c-Jun-N-Terminal Kinase Pathway While Improving Insulin Signaling in Fat-Fed Rats: Evidence for the Role of Oxidative Stress on IRS-1 Serine Phosphorylation and Insulin Resistance. J. Endocrinol..

[35] Abdel-Wahab Y.H., O’Harte F.P., Mooney M.H., Barnett C.R., Flatt P.R. (2002). Vitamin C Supplementation Decreases Insulin Glycation and Improves Glucose Homeostasis in Obese Hyperglycemic (ob/ob) Mice. Metabolism.

[36] Devaraj S., Leonard S., Traber M.G., Jialal I. (2008). Gamma-Tocopherol Supplementation Alone and in Combination with alpha-Tocopherol Alters Biomarkers of Oxidative Stress and Inflammation in Subjects with Metabolic Syndrome. Free. Radic. Biol. Med..

[37] Ashor A.W., Werner A.D., Lara J., Willis N.D., Mathers J.C., Siervo M. (2017). Effects of Vitamin C Supplementation on Glycaemic Control: A Systematic Review and Meta-Analysis of Randomised Controlled Trials. Eur. J. Clin. Nutr..

[38] Kalliora M.I., Vazeou A., Delis D., Bozas E., Thymelli I., Bartsocas C.S. (2011). Seasonal Variation of Type 1 Diabetes Mellitus Diagnosis in Greek Children. Hormones.

[39] Svensson J., Lyngaae-Jørgensen A., Carstensen B., Simonsen L.B., Mortensen H.B. (2009). Long-Term Trends in the Incidence of Type 1 Diabetes in Denmark: The Seasonal Variation Changes over Time. Pediatr. Diabetes.

[40] Wu J., Shao B., Xin X., Luo W., Mo M., Jiang W., Si S., Wang S., Shen Y., Yu Y. (2021). Association of Vitamin D Pathway Gene Polymorphisms with Vitamin D Level during Pregnancy Was Modified by Season and Vitamin D Supplement. Clin. Nutr..

[41] Wei Z., Yoshihara E., He N., Hah N., Fan W., Pinto A.F.M., Huddy T., Wang Y., Ross B., Estepa G. (2018). Vitamin D Switches BAF Complexes to Protect β Cells. Cell.

[42] Mendes A.K.B., Sulis P.M., Cavalari F.C., Padilla D.P.R., Aragón M., Gaspar J.M., Silva F. (2022). 1α,25-(OH)(2) Vitamin D(3) Prevents Insulin Resistance and Regulates Coordinated Exocytosis and Insulin Secretion. J. Nutr. Biochem..

[43] Panjiyar R.P., Dayal D., Attri S.V., Sachdeva N., Sharma R., Bhalla A.K. (2018). Sustained Serum 25-Hydroxyvitamin D Concentrations for One Year with Cholecalciferol Supplementation Improves Glycaemic Control and Slows the Decline of Residual β Cell Function in Children with Type 1 Diabetes. Pediatr. Endocrinol. Diabetes Metab..

[44] Sharma S., Biswal N., Bethou A., Rajappa M., Kumar S., Vinayagam V. (2017). Does Vitamin D Supplementation Improve Glycaemic Control in Children with Type 1 Diabetes Mellitus?—A Randomized Controlled Trial. J. Clin. Diagn. Res..

[45] Pittas A.G., Dawson-Hughes B., Sheehan P., Ware J.H., Knowler W.C., Aroda V.R., Brodsky I., Ceglia L., Chadha C., Chatterjee R. (2019). Vitamin D Supplementation and Prevention of Type 2 Diabetes. N. Engl. J. Med..

[46] Norman A.W., Frankel J.B., Heldt A.M., Grodsky G.M. (1980). Vitamin D Deficiency Inhibits Pancreatic Secretion of Insulin. Science.

[47] Lemieux P., Weisnagel S.J., Caron A.Z., Julien A.S., Morisset A.S., Carreau A.M., Poirier J., Tchernof A., Robitaille J., Bergeron J. (2019). Effects of 6-Month Vitamin D Supplementation on Insulin Sensitivity and Secretion: A Randomised, Placebo-Controlled Trial. Eur. J. Endocrinol..

[48] Juanola-Falgarona M., Salas-Salvadó J., Estruch R., Portillo M.P., Casas R., Miranda J., Martínez-González M.A., Bulló M. (2013). Association between Dietary Phylloquinone Intake and Peripheral Metabolic Risk Markers Related to Insulin Resistance and Diabetes in Elderly Subjects at High Cardiovascular Risk. Cardiovasc. Diabetol..

[49] Yoshida M., Booth S.L., Meigs J.B., Saltzman E., Jacques P.F. (2008). Phylloquinone Intake, Insulin Sensitivity, and Glycemic Status in Men and Women. Am. J. Clin. Nutr..

[50] Beulens J.W., van der A A.D., Grobbee D.E., Sluijs I., Spijkerman A.M., van der Schouw Y.T. (2010). Dietary Phylloquinone and Menaquinones Intakes and Risk of Type 2 Diabetes. Diabetes Care.

[51] Sakamoto N., Nishiike T., Iguchi H., Sakamoto K. (2000). Possible Effects of One Week Vitamin K (Menaquinone-4) Tablets Intake on Glucose Tolerance in Healthy Young Male Volunteers with Different Descarboxy Prothrombin Levels. Clin. Nutr..

[52] Choi H.J., Yu J., Choi H., An J.H., Kim S.W., Park K.S., Jang H.C., Kim S.Y., Shin C.S. (2011). Vitamin K2 Supplementation Improves Insulin Sensitivity via Osteocalcin Metabolism: A Placebo-Controlled Trial. Diabetes Care.

[53] Zwakenberg S.R., Remmelzwaal S., Beulens J.W.J., Booth S.L., Burgess S., Dashti H.S., Imamura F., Feskens E.J.M., van der Schouw Y.T., Sluijs I. (2019). Circulating Phylloquinone Concentrations and Risk of Type 2 Diabetes: A Mendelian Randomization Study. Diabetes.

[54] Ferron M., Hinoi E., Karsenty G., Ducy P. (2008). Osteocalcin Differentially Regulates Beta Cell and Adipocyte Gene Expression and Affects the Development of Metabolic Diseases in Wild-Type Mice. Proc. Natl. Acad. Sci. USA.

[55] Ohsaki Y., Shirakawa H., Miura A., Giriwono P.E., Sato S., Ohashi A., Iribe M., Goto T., Komai M. (2010). Vitamin K Suppresses the Lipopolysaccharide-Induced Expression of Inflammatory Cytokines in Cultured Macrophage-Like Cells via the Inhibition of the Activation of Nuclear Factor κB through the Repression of IKKα/β Phosphorylation. J. Nutr. Biochem..

[56] Sogabe N., Maruyama R., Baba O., Hosoi T., Goseki-Sone M. (2011). Effects of Long-Term Vitamin K(1) (Phylloquinone) or Vitamin K(2) (Menaquinone-4) Supplementation on Body Composition and Serum Parameters in Rats. Bone.

[57] Saeterdal I., Mora J.O., De-Regil L.M. (2012). Fortification of Staple Foods with Vitamin A for Preventing Vitamin A Deficiency.

[58] Wei C., Guoxun C. (2014). The Roles of Vitamin A in the Regulation of Carbohydrate, Lipid, and Protein Metabolism. J. Clin. Med..

[59] Saeed A., Dullaart R.P.F., Schreuder T.C.M.A., Blokzijl H. (2018). Disturbed Vitamin A Metabolism in Non-Alcoholic Fatty Liver Disease (NAFLD). Nutrients.

[60] Kennedy D.O. (2016). B Vitamins and the Brain: Mechanisms, Dose and Efficacy—A Review. Nutrients.

[61] Hanna M., Jaqua E., Nguyen V., Clay J. (2022). B Vitamins: Functions and Uses in Medicine. Perm. J..

[62] Jansen B.C.P., Donath W.F. (1982). Geneeskundig Tijdschrift Voor Nederlandsch-Indie. Nutr. Rev..

[63] Janes R.G., Brady J.M. (1947). Thiamine Deficiency in Adult Normal and Diabetic Rats as Studied under Paired-Feeding Conditions. Fed. Proc..

[64] Beltramo E., Berrone E., Tarallo S., Porta M. (2008). Effects of Thiamine and Benfotiamine on Intracellular Glucose Metabolism and Relevance in the Prevention of Diabetic Complications. Acta Diabetol..

[65] Mascolo E., Vernì F. (2020). Vitamin B6 and Diabetes: Relationship and Molecular Mechanisms. Int. J. Mol. Sci..

[66] Oxenkrug G. (2013). Insulin Resistance and Dysregulation of Tryptophan-Kynurenine and Kynurenine-Nicotinamide Adenine Dinucleotide Metabolic Pathways. Mol. Neurobiol..

[67] Ala O.A., Akintunde A.A., Ikem R.T., Kolawole B.A., Ala O.O., Adedeji T.A. (2017). Association between Insulin Resistance and Total Plasma Homocysteine Levels in Type 2 Diabetes Mellitus Patients in South West Nigeria. Diabetes Metab. Syndr..

[68] Azzini E., Ruggeri S., Polito A. (2020). Homocysteine: Its Possible Emerging Role in At-Risk Population Groups. Int. J. Mol. Sci..

[69] Pratama S., Lauren B.C., Wisnu W. (2022). The Efficacy of Vitamin B(12) Supplementation for Treating Vitamin B(12) Deficiency and Peripheral Neuropathy in Metformin-Treated Type 2 Diabetes Mellitus Patients: A Systematic Review. Diabetes Metab. Syndr..

[70] Liu C., Zhong C., Chen R., Zhou X., Wu J., Han J., Li X., Zhang Y., Gao Q., Xiao M. (2020). Higher Dietary Vitamin C Intake Is Associated with a Lower Risk of Gestational Diabetes Mellitus: A Longitudinal Cohort Study. Clin. Nutr..

[71] Vasudevan S., Hirsch I.B. (2014). Interference of Intravenous Vitamin C with Blood Glucose Testing. Diabetes Care.

[72] Yan M.K., Khalil H. (2017). Vitamin Supplements in Type 2 Diabetes Mellitus Management: A Review. Diabetes Metab. Syndr..

[73] Mason S.A., Rasmussen B., van Loon L.J.C., Salmon J., Wadley G.D. (2019). Ascorbic Acid Supplementation Improves Postprandial Glycaemic Control and Blood Pressure in Individuals with Type 2 Diabetes: Findings of a Randomized Cross-Over Trial. Diabetes Obes. Metab..

[74] Rueangdetnarong H., Sekararithi R., Jaiwongkam T., Kumfu S., Chattipakorn N., Tongsong T., Jatavan P. (2018). Comparisons of the Oxidative Stress Biomarkers Levels in Gestational Diabetes Mellitus (GDM) and Non-GDM among Thai Population: Cohort Study. Endocr. Connect..

[75] Altomare E., Vendemiale G., Chicco D., Procacci V., Cirelli F. (1992). Increased Lipid Peroxidation in Type 2 Poorly Controlled Diabetic Patients. Diabete Metab..

[76] Yao M., Xu F., Yao Y., Wang H., Ju X., Wang L. (2022). Assessment of Novel Oligopeptides from Rapeseed Napin (*Brassica napus*) in Protecting HepG2 Cells from Insulin Resistance and Oxidative Stress. J. Agric. Food Chem..

[77] Hurrle S., Hsu W.H. (2017). The Etiology of Oxidative Stress in Insulin Resistance. Biomed. J..

[78] Balbi M.E., Tonin F.S., Mendes A.M., Borba H.H., Wiens A., Fernandez-Llimos F., Pontarolo R. (2018). Antioxidant Effects of Vitamins in Type 2 Diabetes: A Meta-Analysis of Randomized Controlled Trials. Diabetol. Metab. Syndr..

[79] Paolisso G., D’Amore A., Giugliano D., Ceriello A., Varricchio M., D’Onofrio F. (1993). Pharmacologic Doses of Vitamin E Improve Insulin Action in Healthy Subjects and Non-Insulin-Dependent Diabetic Patients. Am. J. Clin. Nutr..

[80] Paolisso G., D’Amore A., Balbi V., Volpe C., Galzerano D., Giugliano D., Sgambato S., Varricchio M., D’Onofrio F. (1994). Plasma Vitamin C Affects Glucose Homeostasis in Healthy Subjects and in Non-Insulin-Dependent Diabetics. Am. J. Physiol..

[81] Rains J.L., Jain S.K. (2011). Oxidative Stress, Insulin Signaling, and Diabetes. Free Radic. Biol. Med..

[82] Bloch-Damti A., Bashan N. (2005). Proposed Mechanisms for the Induction of Insulin Resistance by Oxidative Stress. Antioxid. Redox Signal..

[83] Liebler D.C., Kaysen K.L., Kennedy T.A. (1989). Redox Cycles of Vitamin E: Hydrolysis and Ascorbic acid Dependent Reduction of 8a-(Alkyldioxy)Tocopherones. Biochemistry.

[84] Tanaka K., Hashimoto T., Tokumaru S., Iguchi H., Kojo S. (1997). Interactions between Vitamin C and Vitamin E Are Observed in Tissues of Inherently Scorbutic Rats. J. Nutr..

[85] Ye Z., Song H. (2008). Antioxidant Vitamins Intake and the Risk of Coronary Heart Disease: Meta-Analysis of Cohort Studies. Eur. J. Cardiovasc. Prev. Rehabil..

[86] Frydrych L.M., Fattahi F., He K., Ward P.A., Delano M.J. (2017). Diabetes and Sepsis: Risk, Recurrence, and Ruination. Front. Endocrinol..

[87] Hongsawong N., Chawprang N., Kittisakmontri K., Vittayananan P., Srisuwan K., Chartapisak W. (2021). Vitamin C Deficiency and Impact of Vitamin C Administration among Pediatric Patients with Advanced Chronic Kidney Disease. Pediatr. Nephrol..

[88] McLean F.C., Budy A.M. (1963). Vitamin A, Vitamin D, Cartilage, Bones, and Teeth. Vitam. Horm..

[89] Norman A.W. (2012). The History of the Discovery of Vitamin D and Its Daughter Steroid Hormone. Ann. Nutr. Metab..

[90] Holick M.F., Schnoes H.K., DeLuca H.F. (1971). Identification of 1,25-Dihydroxycholecalciferol, a Form of Vitamin D3 Metabolically Active in the Intestine. Proc. Natl. Acad. Sci. USA.

[91] Quaresima P., Angeletti M., Luziatelli D., Luziatelli S., Venturella R., Di Carlo C., Bernardo S. (2021). Pregnancy Associated Transient Osteoporosis of the Hip (PR-TOH): A Non-Obstetric Indication to Caesarean Section. A Case Report with Literature Review. Eur. J. Obstet. Gynecol. Reprod. Biol..

[92] Cândido F.G., Bressan J. (2014). Vitamin D: Link between Osteoporosis, Obesity, and Diabetes?. Int. J. Mol. Sci..

[93] Lu L., Bennett D.A., Millwood I.Y., Parish S., McCarthy M.I., Mahajan A., Lin X., Bragg F., Guo Y., Holmes M.V. (2018). Association of Vitamin D with Risk of Type 2 Diabetes: A Mendelian Randomisation Study in European and Chinese Adults. PLoS Med..

[94] Rihal V., Khan H., Kaur A., Singh T.G. (2022). Vitamin D as Therapeutic Modulator in Cerebrovascular Diseases: A Mechanistic Perspectives. Crit. Rev. Food Sci. Nutr..

[95] Li J., Qin S., Zhang S., Lu Y., Shen Q., Cheng L., Zhong R. (2023). Serum Vitamin D Concentration, Vitamin D-Related Polymorphisms, and Colorectal Cancer Risk. Int. J. Cancer.

[96] Dominguez L.J., Farruggia M., Veronese N., Barbagallo M. (2021). Vitamin D Sources, Metabolism, and Deficiency: Available Compounds and Guidelines for Its Treatment. Metabolites.

[97] Christakos S., Dhawan P., Verstuyf A., Verlinden L., Carmeliet G. (2016). Vitamin D: Metabolism, Molecular Mechanism of Action, and Pleiotropic Effects. Physiol. Rev..

[98] Neu A., Kehrer M., Hub R., Ranke M.B. (1997). Incidence of IDDM in German Children Aged 0–14 Years. A 6-Year Population-Based Study (1987–1993). Diabetes Care.

[99] Karvonen M., Jäntti V., Muntoni S., Stabilini M., Stabilini L., Muntoni S., Tuomilehto J. (1998). Comparison of the Seasonal Pattern in the Clinical Onset of IDDM in Finland and Sardinia. Diabetes Care.

[100] Moltchanova E.V., Schreier N., Lammi N., Karvonen M. (2009). Seasonal Variation of Diagnosis of Type 1 Diabetes Mellitus in Children Worldwide. Diabet. Med..

[101] Bailey R., Cooper J.D., Zeitels L., Smyth D.J., Yang J.H., Walker N.M., Hyppönen E., Dunger D.B., Ramos-Lopez E., Badenhoop K. (2007). Association of the Vitamin D Metabolism Gene CYP27B1 with Type 1 Diabetes. Diabetes.

[102] Cooper J.D., Smyth D.J., Walker N.M., Stevens H., Burren O.S., Wallace C., Greissl C., Ramos-Lopez E., Hyppönen E., Dunger D.B. (2011). Inherited Variation in Vitamin D Genes is Associated with Predisposition to Autoimmune Disease Type 1 Diabetes. Diabetes.

[103] Nejentsev S., Cooper J.D., Godfrey L., Howson J.M., Rance H., Nutland S., Walker N.M., Guja C., Ionescu-Tirgovişte C., Savage D.A. (2004). Analysis of the Vitamin D Receptor Gene Sequence Variants in Type 1 Diabetes. Diabetes.

[104] Pittas A.G., Kawahara T., Jorde R., Dawson-Hughes B., Vickery E.M., Angellotti E., Nelson J., Trikalinos T.A., Balk E.M. (2023). Vitamin D and Risk for Type 2 Diabetes in People with Prediabetes: A Systematic Review and Meta-Analysis of Individual Participant Data from 3 Randomized Clinical Trials. Ann. Intern. Med..

[105] Van Belle T.L., Gysemans C., Mathieu C. (2013). Vitamin D and diabetes: The Odd Couple. Trends Endocrinol. Metab..

[106] Cade C., Norman A.W. (1987). Rapid Normalization/Stimulation by 1,25-Dihydroxyvitamin D3 of Insulin Secretion and Glucose Tolerance in the Vitamin D-Deficient Rat. Endocrinology.

[107] Rasouli N., Brodsky I.G., Chatterjee R., Kim S.H., Pratley R.E., Staten M.A., Pittas A.G. (2022). Effects of Vitamin D Supplementation on Insulin Sensitivity and Secretion in Prediabetes. J. Clin. Endocrinol. Metab..

[108] Provvedini D.M., Tsoukas C.D., Deftos L.J., Manolagas S.C. (1983). 1,25-Dihydroxyvitamin D3 Receptors in Human Leukocytes. Science.

[109] Provvedini D.M., Tsoukas C.D., Deftos L.J., Manolagas S.C. (1986). 1 Alpha,25-Dihydroxyvitamin D3-Binding Macromolecules in Human B Lymphocytes: Effects on Immunoglobulin Production. J. Immunol..

[110] Veldman C.M., Cantorna M.T., DeLuca H.F. (2000). Expression of 1,25-Dihydroxyvitamin D(3) Receptor in the Immune System. Arch. Biochem. Biophys..

[111] Wolden-Kirk H., Overbergh L., Christesen H.T., Brusgaard K., Mathieu C. (2011). Vitamin D and Diabetes: Its Importance for Beta Cell and Immune Function. Mol. Cell Endocrinol..

[112] Fusaro M., Gallieni M., Porta C., Nickolas T.L., Khairallah P. (2020). Vitamin K Effects in Human Health: New Insights beyond Bone and Cardiovascular Health. J. Nephrol..

[113] Ellis J.L., Karl J.P., Oliverio A.M., Fu X., Soares J.W., Wolfe B.E., Hernandez C.J., Mason J.B., Booth S.L. (2021). Dietary Vitamin K Is Remodeled by Gut Microbiota and Influences Community Composition. Gut Microbes.

[114] Zhang S., Guo L., Bu C. (2019). Vitamin K Status and Cardiovascular Events or Mortality: A Meta-Analysis. Eur. J. Prev. Cardiol..

[115] Vervoort L.M., Ronden J.E., Thijssen H.H. (1997). The Potent Antioxidant Activity of the Vitamin K Cycle in Microsomal Lipid Peroxidation. Biochem. Pharmacol..

[116] Dihingia A., Ozah D., Ghosh S., Sarkar A., Baruah P.K., Kalita J., Sil P.C., Manna P. (2018). Vitamin K1 Inversely Correlates with Glycemia and Insulin Resistance in Patients with Type 2 Diabetes (T2D) and Positively Regulates SIRT1/AMPK Pathway of Glucose Metabolism in Liver of T2D Mice and Hepatocytes Cultured in High Glucose. J. Nutr. Biochem..

[117] Asadipooya K., Graves L., Lukert B.P., Kalantarhormozi M., Assadi M., Ostovar A., Larijani B., Nabipour I. (2015). Osteocalcin Is a Predictor for Diabetes Mellitus in Postmenopausal Women and Correlated with Oral Intake of Vitamin K. Mediterr. J. Nutr. Metab..

[118] Lee N.K., Sowa H., Hinoi E., Ferron M., Ahn J.D., Confavreux C., Dacquin R., Mee P.J., McKee M.D., Jung D.Y. (2007). Endocrine Regulation of Energy Metabolism by the Skeleton. Cell.

[119] Ibarrola-Jurado N., Salas-Salvadó J., Martínez-González M.A., Bulló M. (2012). Dietary Phylloquinone Intake and Risk of Type 2 Diabetes in Elderly Subjects at High Risk of Cardiovascular Disease. Am. J. Clin. Nutr..

[120] Rasekhi H., Karandish M., Jalali M.T., Mohammadshahi M., Zarei M., Saki A., Shahbazian H. (2015). Phylloquinone Supplementation Improves Glycemic Status Independent of the Effects of Adiponectin Levels in Premonopause Women with Prediabetes: A Double-Blind Randomized Controlled Clinical Trial. J. Diabetes Metab. Disord..

[121] Rasekhi H., Karandish M., Jalali M.T., Mohammad-Shahi M., Zarei M., Saki A., Shahbazian H. (2015). The Effect of Vitamin K1 Supplementation on Sensitivity and Insulin Resistance via Osteocalcin in Prediabetic Women: A Double-Blind Randomized Controlled Clinical Trial. Eur. J. Clin. Nutr..

[122] Assimacopoulos-Jeannet F. (2004). Fat Storage in Pancreas and in Insulin-Sensitive Tissues in Pathogenesis of Type 2 Diabetes. Int. J. Obes. Relat. Metab. Disord..

[123] Kumar R., Binkley N., Vella A. (2010). Effect of Phylloquinone Supplementation on Glucose Homeostasis in Humans. Am. J. Clin. Nutr..

